# An investigation of the stirring duration effect on synthesized graphene oxide for dye-sensitized solar cells

**DOI:** 10.1371/journal.pone.0228322

**Published:** 2020-02-03

**Authors:** Xin Hui Yau, Foo Wah Low, Cheng Seong Khe, Chin Wei Lai, Sieh Kiong Tiong, Nowshad Amin

**Affiliations:** 1 Department of Fundamental and Applied Sciences, Universiti Teknologi PETRONAS, Seri Iskandar, Perak, Malaysia; 2 Centre of Innovative Nanostructures and Nanodevices (COINN), Universiti Teknologi PETRONAS, Seri Iskandar, Perak, Malaysia; 3 Institute of Sustainable Energy, Universiti Tenaga Nasional (The National Energy University), Jalan IKRAM-UNITEN, Kajang, Selangor, Malaysia; 4 Nanotechnology & Catalysis Research Centre (NANOCAT), Institute of Postgraduate Studies (IPS), University of Malaya, Kuala Lumpur, Malaysia; University of California Santa Barbara, California, USA, INDIA

## Abstract

This study investigates the effects of stirring duration on the synthesis of graphene oxide (GO) using an improved Hummers’ method. Various samples are examined under different stirring durations (20, 40, 60, 72, and 80 h). The synthesized GO samples are evaluated through X-ray diffraction (XRD), field emission scanning electron microscopy (FESEM), energy dispersive spectroscopy (EDX), Fourier transform infrared spectroscopy (FTIR), and Raman spectroscopy. The GO sample with 72 h stirring duration (GO72) has the highest d-spacing in the XRD results, highest atomic percentage of oxygen in EDX (49.57%), highest intensity of oxygen functional group in FTIR spectra, and highest intensity ratio in Raman analysis (I_D_/I_G_ = 0.756). Results show that GO72 with continuous stirring has the highest degree of oxidation among other samples. Electrochemical impedance spectroscopy analysis shows that GO72–titanium dioxide (TiO_2_) exhibits smaller charge transfer resistance and higher electron lifetime compared with the TiO_2_-based photoanode. The GO72 sample incorporating TiO_2_ nanocomposites achieves 6.25% photoconversion efficiency, indicating an increase of more than twice than that of the mesoporous TiO_2_ sample. This condition is fully attributed to the efficient absorption rate of nanocomposites and the reduction of the recombination rate of TiO_2_ by GO in dye-sensitized solar cells.

## Introduction

Graphene is a monolayer sp^2^ carbon atoms tightly packed in a 2D honeycomb lattice [1]. Graphene has been widely investigated because of its outstanding properties, such as high electron mobility (20 m^2^/vs), high thermal conductivity (5000 W/mK), and optimum mechanical properties (1.0 TPa) [1, 2]. Graphene shows promising potential in various applications, such as biosensors [3], catalysis [4], photovoltaics [5, 6], and etc [7].

Graphene can be obtained through several methods, including chemical vapor deposition, liquid phase exfoliation, and mechanical exfoliation of graphite [8, 9]. Graphene oxide (GO) reduction is another alternative to obtain graphene at low cost (graphite powder is used as starting material to obtain GO) and in bulk quantities. GO is the oxidized form of graphene. GO is composed of oxygen functional groups, such as epoxyl and hydroxyl groups at the basal plane, and carbonyl and carboxyl groups at the edge of graphene sheet. These oxygen functional groups enables GO to exhibit good hydrophilicity, allowing it to disperse well in solvents, especially water [10, 11].

In this study, the effect of stirring duration on GO synthesized using an improved Hummers’ method was investigated. Stirring durations of 20, 40, 60, 72, and 80 h were examined to determine the optimum stirring duration for synthesizing GO with high degree oxidation. The characteristic differences of the obtained samples were studied through X-ray diffraction (XRD), field emission scanning electron microscopy (FESEM), electron dispersive spectroscopy (EDX), Fourier transform infrared spectroscopy (FTIR), and Raman spectroscopy. The optimized GO sample was incorporated with TiO_2_ as composite materials for photoanode to increase the absorption rate of excited dye in dye-sensitized solar cells (DSSCs).

## Experimental section

### Materials

Graphite powder (< 45 μm; ≥ 99.99%), fluorine-doped tin oxide-coated glass (FTO) slide with surface resistivity of ~7 Ω/sq, and di-tetrabutylammonium *cis*-bis (isothiocyanato) bis (2,2'-bipyridyl- 4,4'dicarboxylato) ruthenium(II), and N-719 dye were purchased from Sigma-Aldrich, Malaysia. Sulfuric acid (H_2_SO_4_, 98%), phosphoric acid (H_3_PO_4_, 85%), potassium permanganate (KMnO_4_, 99.9%), hydrogen peroxide (H_2_O_2_, 30%), hydrochloric acid (HCl, 37%), absolute ethanol (≥ 99.5%), acetonitrile (C_2_H_3_N; 41.05 g/mol), and potassium iodide (KI; ≥ 99.0%) electrolyte were purchased from Merck, Malaysia. All chemicals were used without purification. Deionized water was used in all experiments. The applied metal oxide of synthesized TiO_2_ was previously reported in our work [12].

### GO synthesis

GO was synthesized from graphite powders using an improved Hummers’ method. KMnO_4_ (9.0 g) was added with H_2_SO_4_ (360 mL) and H_3_PO_4_ (40 mL). Graphite powder (3.0 g) was gradually added to the mixture. The mixture was then stirred under ice bath condition (< 20°C) for 1 h. Then, the ice bath was discarded and replaced with water bath for the subsequent experiment. The mixture was stirred at different stirring times (20, 40, 60, 72, and 80 h) with constant speed (500 rpm) and maintained at 45°C to ensure complete oxidation of graphite. At the end of stirring time, the mixture was poured with 400 g of ice cubes (produced from deionized water), and H_2_O_2_ was then added subsequently to stop oxidation. The mixture was left for two weeks for the settlement of solid. The solid was first washed with 1 M of HCl and left overnight for settlement. The settlement was washed with deionized water through centrifugation until pH 4–5 was achieved. The resulting settlements (GO) obtained were dried in an oven at 40°C overnight to form GO solid.

### Preparation of DSSCs

**Fig 1:**
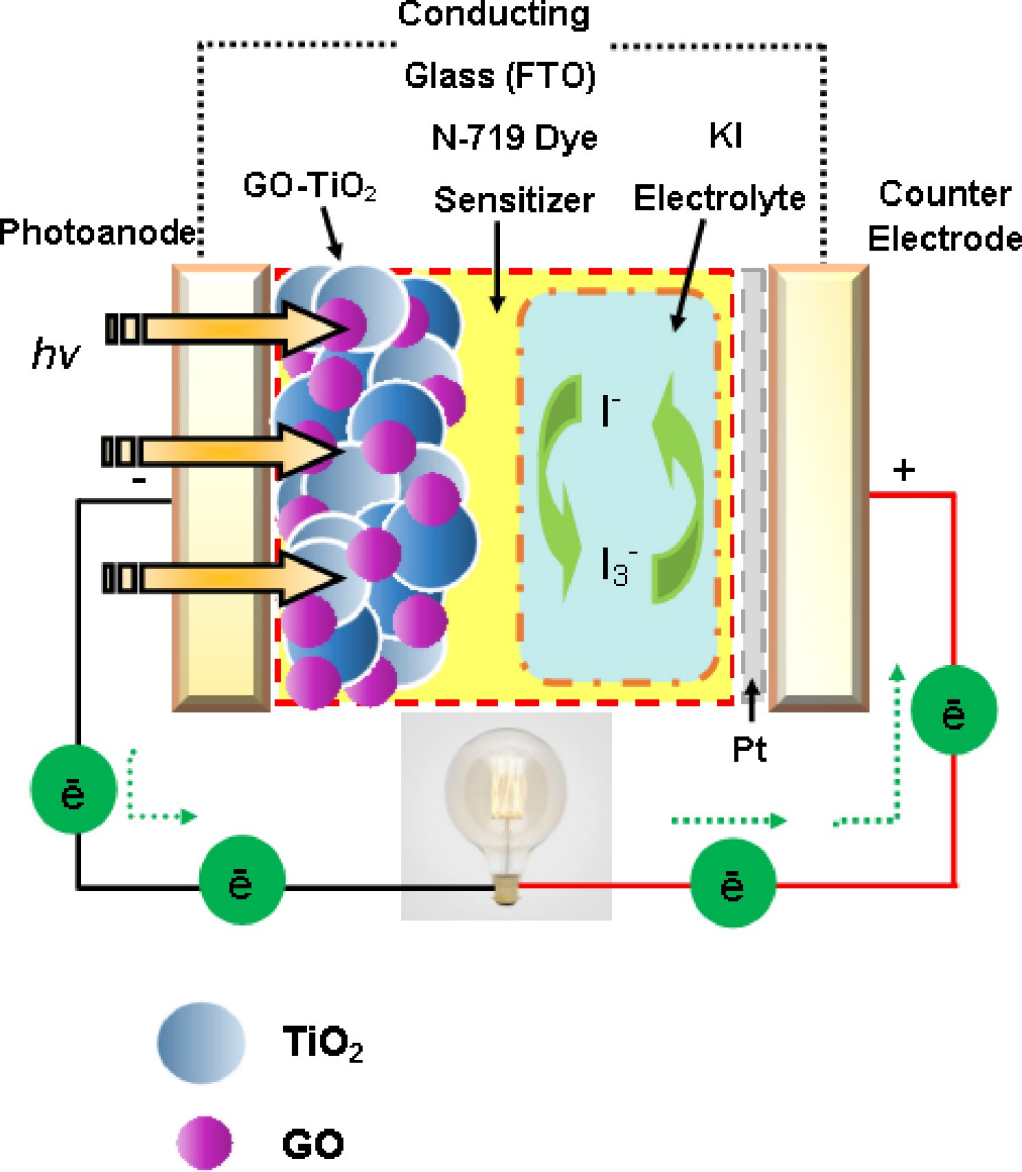
Schematic of GO72–TiO_2_ nanocomposite-based photoanode in DSSCs.

The structure of TiO_2_ incorporated with GO (GO72–TiO_2_) nanocomposite-based photoanode for DSSCs is shown in Fig 1. The entire interface of DSSCs were assembled in sandwiched configuration, which are composed of FTO glass slide as photoanode for allowing visible light absorption, N-719 dye sensitizer for electrostatic binding with TiO_2_, KI electrolyte for redox pair reaction and counter electrode for carrier electrons from the outer circuit and regenerating excitation/oxidation/reduction. FTO glasses were cut into small pieces with dimensions of 2 cm × 2 cm, and the active area for the device was 0.67 cm^2^. For the fabrication of DSSCs, the FTO-coated TiO_2_ was immersed overnight in a solvent containing 0.5 Mm N-719 and absolute ethanol. The same process was performed for the FTO-coated GO72–TiO_2_ sample. The photoanodes were rinsed with acetonitrile to remove the physiosorbed N-719 dye molecules and dried on a hot plate for 10 min. Each FTO glass slide was sintered at 450°C for 15 min. The counter electrode was deposited with Pt thin film through sputtering under 266.64 mPa constant pressure, 0.67 mPa base pressure, 150 W sputter power, 15 mL Ar gas flow rate, and 60 s sputter duration. Consequently, the photoanodes containing N-719 based TiO_2_ and GO72–TiO_2_ were sandwiched with counter electrode and clipped with a paper clip. KI electrolyte (0.5 M) was applied between the electrodes.

### Characterization

The crystalline structure of GO was identified using a Bruker D8 advance diffractometer (XRD) with Cu Kα radiation. The surface morphology of GO was studied using a ZEISS Leo Supra 55 variable pressure field emission scanning electron microscope (FESEM). The surface bonding of the particles was recorded with a Perkin Elmer Spectrum BX Fourier transform infrared spectrophotometer (FTIR) in the range of 500–4000 cm^−1^ using KBr pellet method. Raman spectrum was performed using a Horiba Jobin-Yvon HR800 Raman spectrometer equipped with a 514.5 nm laser source to characterize GO. For the photovoltaic performance test, the measurements of photocurrent density versus applied voltage (*J–V*) curves was obtained under AM 1.5 condition using an AUT 50284, Metrohm Autolab instrument (PGSTAT204). The electrochemical impedance spectroscopy (EIS) results were analyzed using a Metrohm Autolab instrument (PGSTAT100N).

## Results and discussion

GO samples were synthesized from graphite powder using an improved Hummers’ method. During synthesis, the mixture color changed from dark purple green to dark brown when graphite was added to the mixture (KMnO_4_, H_2_SO_4_, and H_3_PO_4_). This condition showed that graphite was fully mixed with chemical reagents during oxidation. The mixture color changed to light brown when H_2_O_2_ was introduced to stop the oxidation, indicating a high oxidation degree of graphite [13]. In this study, the effects of stirring duration with a constant speed (500 rpm) on the formation of GO were studied, as shown in Table 1. The main objective was to determine the optimum stirring duration for obtaining GO with high oxidation ratios.

**Table 1:** Synthesis parameter of GO.

GO Sample	Stirring Duration (hours)
GO20	20
GO40	40
GO60	60
GO72	72
GO80	80

**Fig 2:**
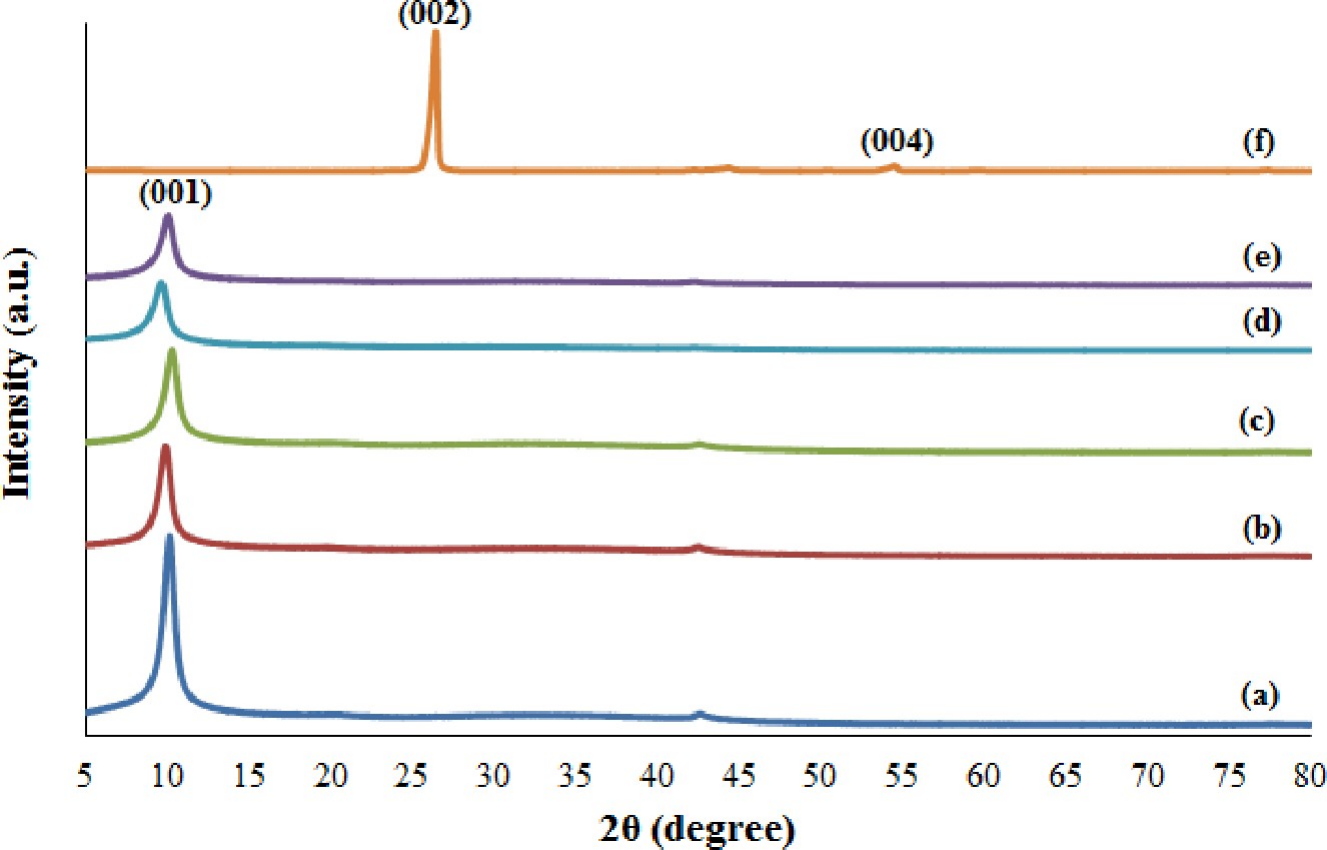
XRD patterns of (a) GO20, (b) GO40, (c) GO60, (d) GO72, (e) GO80, and (f) graphite.

In this experiment, XRD analysis was used as reference to identify the sample and to determine its crystallite size. XRD was also used to study the degree of graphite oxidation under different stirring durations on the basis of interlaminar distance (also known as d-spacing). The XRD patterns of GO20, GO40, GO60, GO72, GO80, and graphite are shown in Fig 2. The XRD analysis results (including peak position, d-spacing, FWHM and crystallite size) of GO and graphite samples are shown in Table 2. As shown in (Fig 2A–2E), GO (GO20, GO40, GO60, GO72 and GO80) shows an intense (001) peak at 2θ ≈ 10°–11°, which is the characteristic of GO [14]. As shown in Fig 2(F), graphite displays an intense (002) peak at 2θ = 26.24^o^, which is similar in literature [14]. Graphite (starting material) peak disappears at 2θ = 26.24°, and GO (final product) peaks appear at 2θ ≈ 10°–11°, indicating that graphite is fully oxidized to GO. As shown in (Fig 2A–2C), GO20, GO40, and GO60 have a weak peak at 2θ = 41.78°, indicating the turbostratic band of disordered carbon materials [15]. As shown in Fig 2(F), a weak (004) peak is observed at 2θ = 54.45°, indicating a Brag reflection of graphite phase [13].

**Table 2:** Peak position, d-spacing, FWHM, and crystallite size of GO and graphite.

Sample	Peak Position (^o^)	d-spacing (Å)	FWHM	Crystallite Size (nm)
GO20	10.78	8.20	0.8141	10.23
GO40	9.91	8.91	0.8929	9.32
GO60	10.85	8.14	0.7484	11.13
GO72	9.60	9.20	0.9848	8.45
GO80	10.05	8.79	0.7353	11.32
Graphite	26.24	3.39	0.0263	323.82

The oxidation degree of graphite can be determined by calculating the d-spacing using Bragg’s Law [16], which can be expressed as:

$$n\lambda =2d_{hkl}sin\theta ,$$ (1)

where n is an integer representing the order of diffraction, λ is the wavelength of X-ray (λ = 0.15406 nm), d_hkl_ is the interplanar distance or d-spacing of Miller indices, and θ is the scattering angle. The d-spacing of GO (8.14 Å–9.20 Å), is higher compared with the d-spacing of graphite (3.39 Å) regardless of the stirring duration. This condition is because of the introduction of the oxygenated functional groups, such as carbonyl, carboxyl, epoxyl and hydroxyl to the graphite sheets during oxidation, thereby expanding the interlayer spacing between them. The crystallite size of the sample can be calculated using the Debye-Scherrer equation [17] as follows:

$$D=\frac{K\lambda }{\beta \;\cos\;\theta },$$ (2)

where D is the crystallite size of the sample, K is the Scherrer constant (0.9), λ is the X-ray wavelength (0.15406 nm), β is the width of XRD peak at half height (FWHM), and θ is the Bragg diffraction angle. The crystallite size of GO ranges from 8.45 nm to 11.32 nm regardless of the stirring duration. These crystallite sizes are smaller compared with graphite (323.82 nm) because oxidation can break down the crystallite. GO72 is the best sample because it has the highest d-spacing value (many oxygen functional groups intercalate with the graphite sheets) and the smallest crystallite size (large surface area for intercalation of oxygen functional groups).

**Fig 3:**
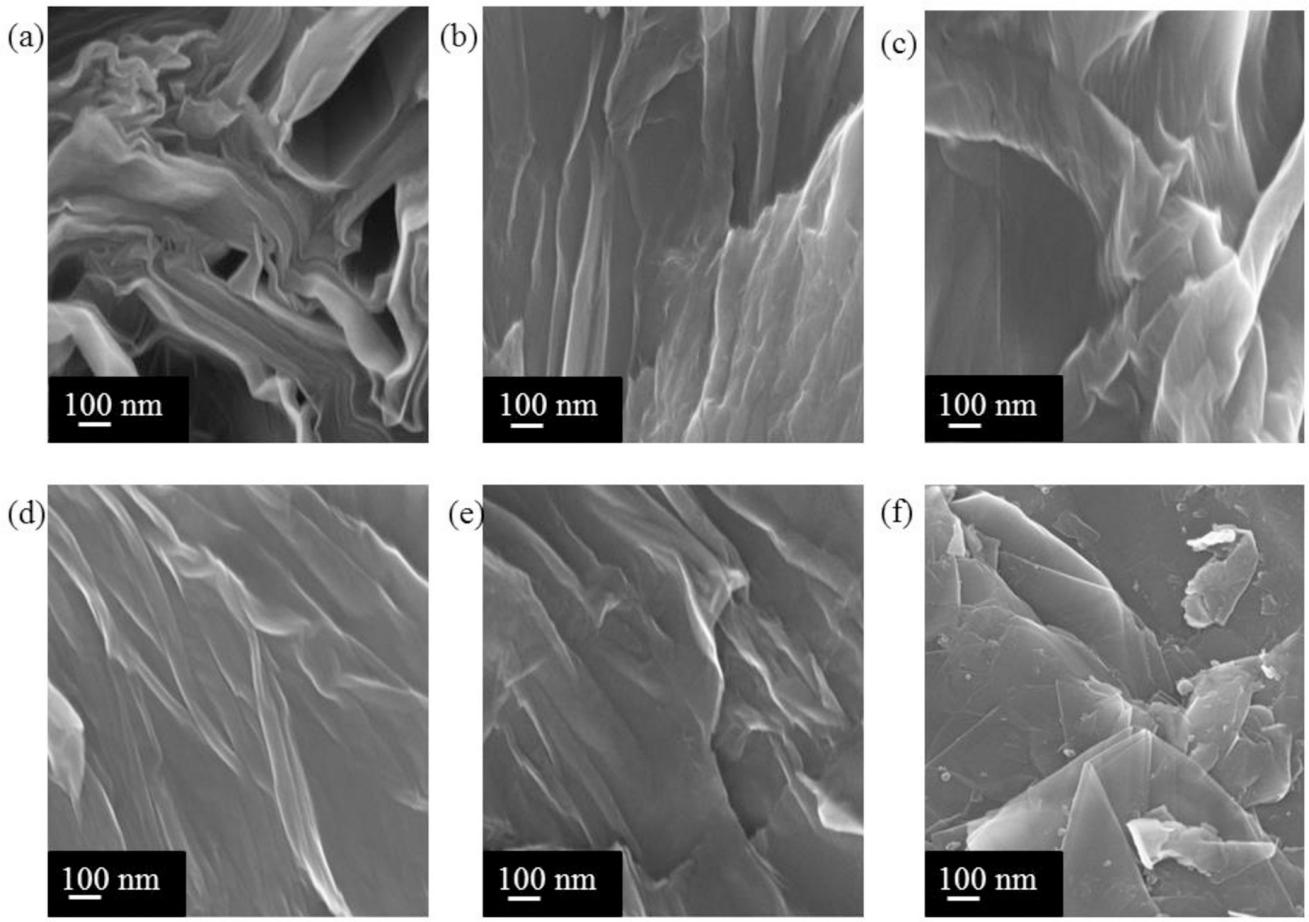
FESEM of (a) GO20, (b) GO40, (c) GO60, (d) GO72, (e) GO80, and (f) graphite.

The morphology of GO and graphite was studied through FESEM. As shown in (Fig 3A–3E), GO exhibits multiple layer structures. Many GOs are exfoliated, and GO with few layers are obtained with the increase in stirring duration. This condition agrees with the XRD results, where GO obtained with 72 h exhibits the smallest crystallite size. This condition is attributed to the strong acidic environment combined with mechanical agitation, thereby decomposing the graphite particles into small pieces and exfoliating the sheets. As shown in (Fig 3A–3E), GO also exhibits wrinkled structure that causes sheet folding. This condition is because the GO sheet is loaded with many oxygen-containing functional groups that are mainly distributed at the edges of the GO sheet [16]. As shown in Fig 3(F), graphite is shown in a platelet-liked crystalline form of carbon, which is similar to literature [13].

**Fig 4:**
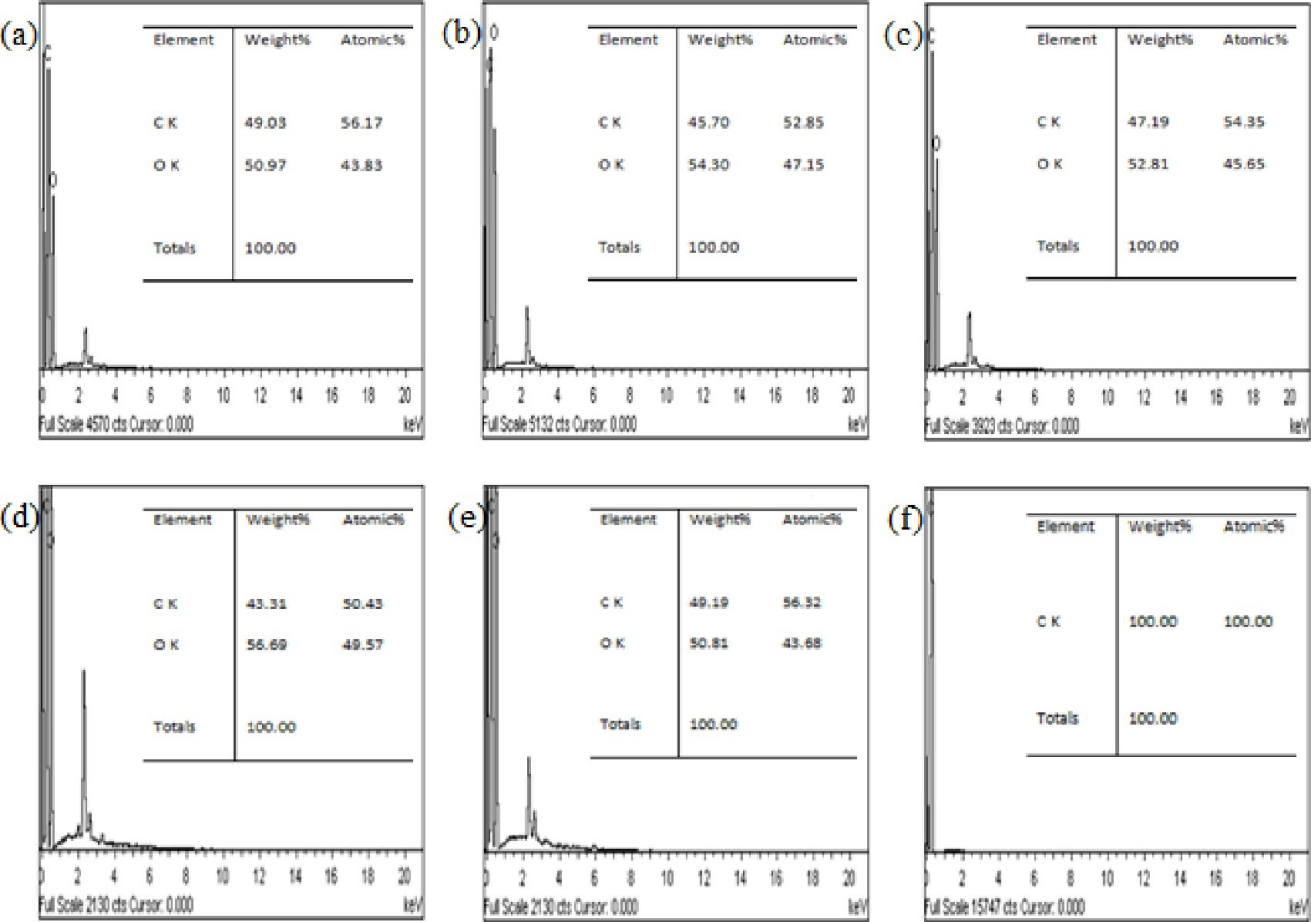
EDX of (a) GO20, (b) GO40, (c) GO60, (d) GO72, (e) GO80, and (f) graphite.

EDX analysis was conducted to investigate the atomic percentage of carbon and oxygen in the synthesized GO samples. (Fig 4A–4E) show the carbon and oxygen contents of GO, whereas Fig 4(F) only shows the carbon content of graphite. Table 3 shows the summarized data of EDX analysis of GO and graphite in terms of carbon content (atomic %), oxygen content (in atomic %), and the ratio of carbon to oxygen composition. As shown in Fig 4(F), graphite powder contains 100% carbon, indicating the purity of graphite powder as the starting material. GO (regardless of stirring duration) contains approximately 50.43% to 56.32% and 43.68% to 49.57% of carbon and oxygen contents, respectively. Compared with graphite, carbon content decreases, whereas oxygen content increases in GO, indicating that chemical oxidation occurs when carbon is replaced by oxygenated groups after oxidation. GO72 is the best oxidized sample because it contains the lowest carbon content (50.43%), highest oxygen content (49.57%), and lowest C/O ratio (C/O = 1.0173). This result suggests that GO72 has the best stirring duration to obtain GO with high oxidation degree.

**Table 3:** Summary of EDX analysis (atomic %) of GO and graphite.

Sample	C (%)	O (%)	C/O ratio
GO20	56.17	43.83	1.2815
GO40	52.85	47.15	1.1209
GO60	54.35	45.65	1.1906
GO72	50.43	49.57	1.0173
GO80	56.32	43.68	1.2894
Graphite	100.00	0.00	N/A

**Fig 5:**
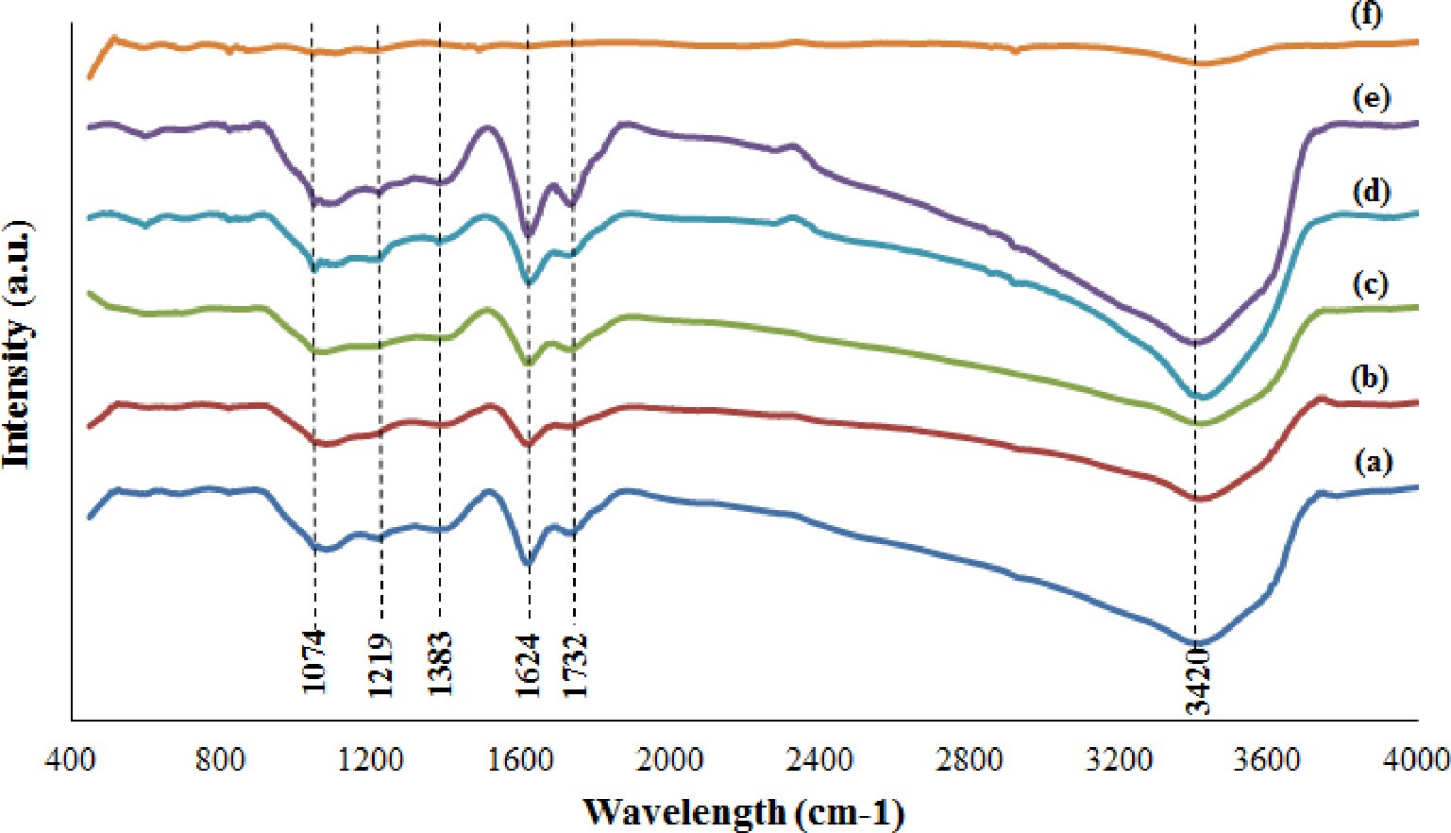
FTIR spectra of (a) GO20, (b) GO40, (c) GO60, (d) GO72, (e) GO80, and (f) graphite.

FTIR analysis was used to determine the surface functional group of the sample. (Fig 5A–5E) and 5(F) show the FTIR spectra of GO and graphite. The FTIR spectrum of graphite (Fig 5(F)) is mostly composed of carbon, and no oxygen bonds are observed. This condition is consistent with the work demonstrated by Lavin-Lopez et al. [18]. By contrast, the FTIR spectra of all GO samples (regardless of stirring duration) in (Fig 5A–5E) exhibit similar characteristic peaks at 1074, 1219, 1383, 1624, 1732 and 3420 cm^−1^, which are assigned to anhydride group CO-O-CO, epoxyl C-O, carbonyl C = O, aromatic C = C stretching, carboxyl O = C-OH, and hydroxyl–OH groups, respectively [18–20]. All these findings prove that the oxygen functional groups are successfully introduced to the graphite surface after oxidation under the presence of KMnO_4_ and H_2_SO_4_. GO72 shows a relatively high intensity of FTIR spectra compared with others, thereby indicating that the highest oxidation degree of the GO sample can be obtained through 72 h continuous stirring.

**Fig 6:**
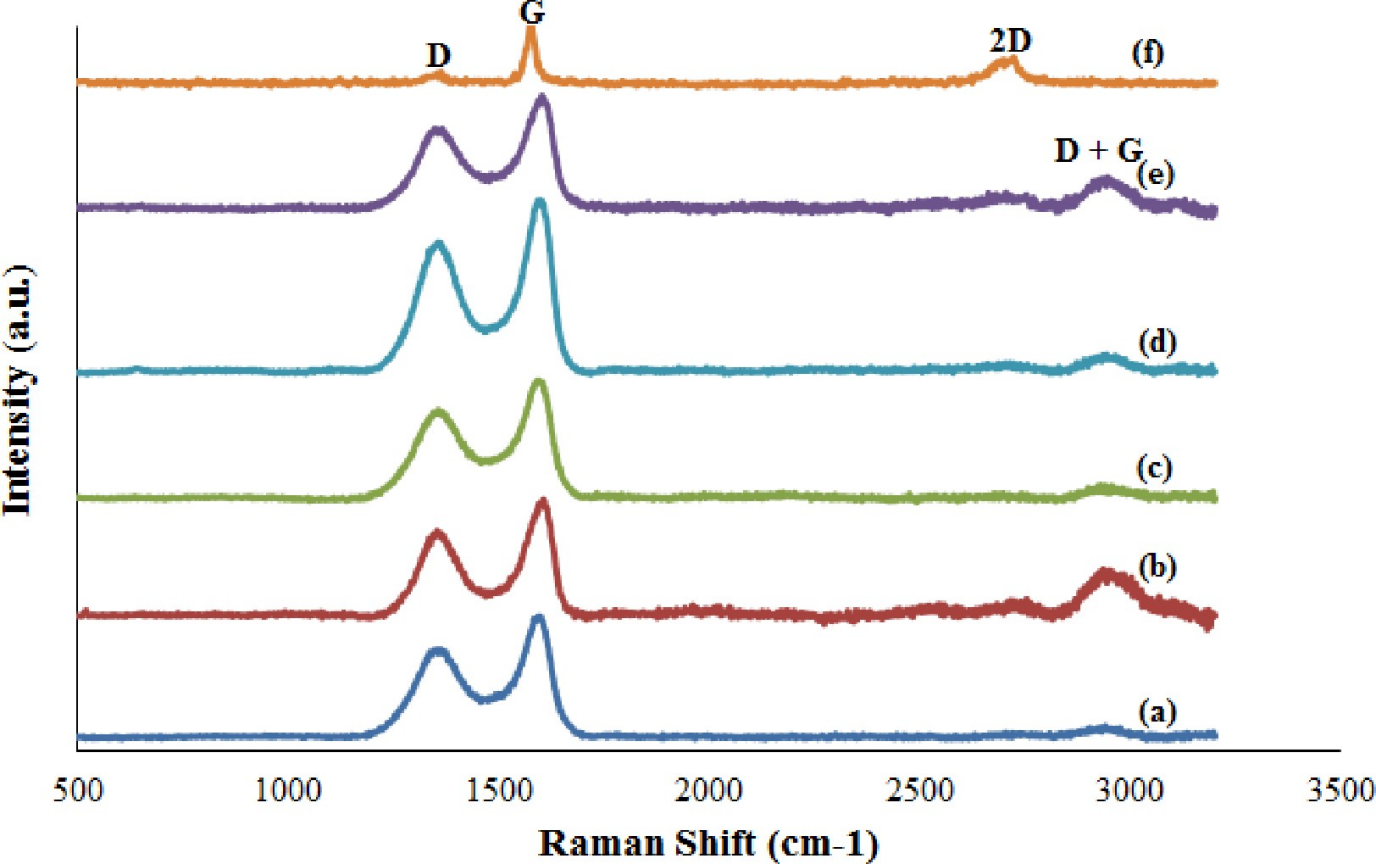
Raman spectra of (a) GO20, (b) GO40, (c) GO60, (d) GO72, (e) GO80, and (f) graphite.

Raman spectroscopy is a powerful tool used to characterize carbon-based materials. (Fig 6A–6E) and 6(F) show the Raman spectra of GO and graphite. Table 4 shows the summarized data of Raman analysis of GO and graphite in terms of the D band, G band, 2D band, D + G band, and the intensity ratio of D and G bands (I_D_/I_G_). As shown in (Fig 6A–6E), GO consists of two prominent peaks located at (1347.77 cm^−1^ to 1352.37 cm^−1^) and (1597.40 cm^−1^ to 1603.38 cm^−1^), which corresponds to the D and G bands, respectively [21], and one D + G band (2942.06 cm^−1^ to 2948.32 cm^−1^) [16]. For the Raman spectrum of graphite (Fig 6(F)), the peaks of D, G, and 2D bands are located at 1352.37, 1601.87 and 2701.80 cm^−1^. The D band refers to the disorder mode caused by the oxidation of graphite, whereas the G band represents the first-order E_2g_ mode from the sp^2^ carbon domain [22]. The 2D band, also known as the G’ band, is the second prominent band in the Raman spectrum of graphite sample. The D + G band is a defect-activated peak [16], and its presence in the Raman spectra of GO indicates that the introduction of oxygen functional groups causes some structural defects in GO. The intensity ratio of D and G bands (I_D_/I_G_) was measured to determine the degree of disorder [23]. GO72 shows the highest intensity ratio compared with others. This result indicated that the GO72 stirring duration experiences many defects during oxidation and exfoliation process. In other words, many oxygen functional groups are introduced at the basal plane and the edges of GO, which is synthesized with 72 h continuous stirring.

**Table 4:** Raman analysis of GO and graphite.

Sample	D (cm^-1^)	G (cm^-1^)	2D (cm^-1^)	D + G (cm^-1^)	I_D_/I_G_ ratio
GO20	1347.77	1597.40	N/A	2943.31	0.722
GO40	1352.37	1603.36	N/A	2948.32	0.734
GO60	1352.37	1603.36	N/A	2942.06	0.754
GO72	1352.37	1600.38	N/A	2944.56	0.756
GO80	1352.37	1601.87	N/A	2947.07	0.712
Graphite	1353.96	1572.65	2701.80	N/A	0.211

**Fig 7:**
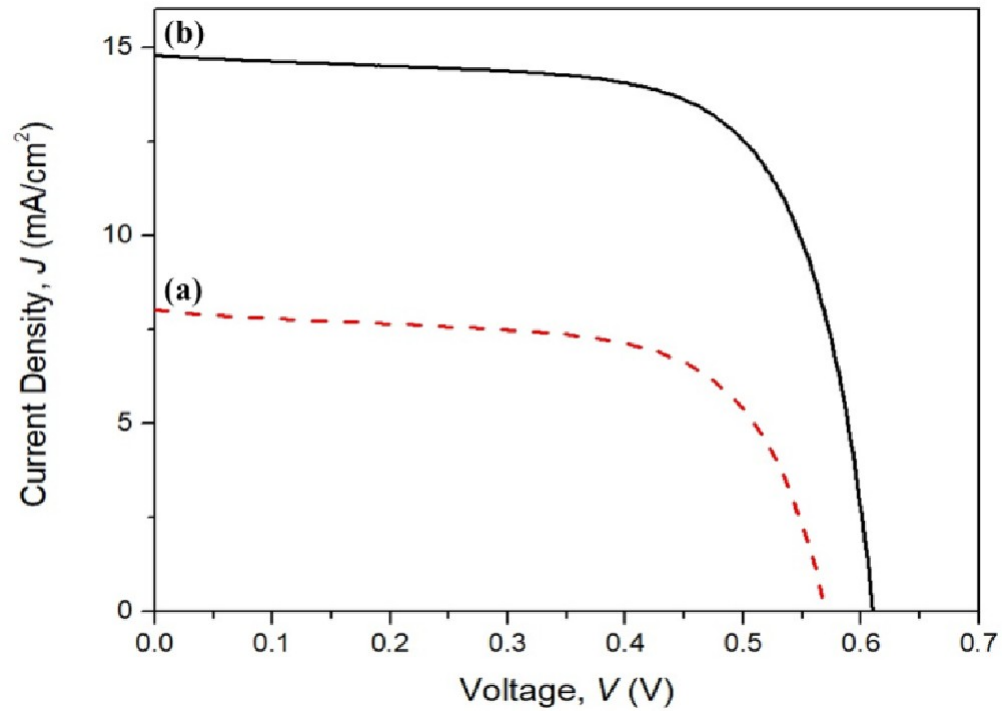
*J–V* curves of DSSCs under AM 1.5 condition (power density, *Px* = 100 mW/cm^2^) with photoanode based on (a) mesoporous TiO_2_ and (b) GO72–TiO_2_ nanocomposite.

For the DSSCs, GO72 was selected as the composite material for testing because of its good integral properties. Fig 7 shows the current density versus voltage *J–V* characteristic curve of DSSCs with different photoanode materials based on mesoporous TiO_2_ and GO72–TiO_2._ The comprehensive parameters are tabulated in Table 5. The short circuit current density *Jsc* of GO72–TiO_2_ nanocomposite could achieve double than that of mesoporous TiO_2_ (from 8.02 mA/cm^2^ to 14.78 mA/cm^2^), and the photoconversion efficiency (PCE) significantly increases from 2.96% to 6.25% (~111% increased PCE), which is probably attributed to the enhancement of photon loading absorption under visible light condition [24]. In other words, GO72 can be used as charge carrier facilitator agent to allow frequent flow of electrons from the conduction band (CB) of TiO_2_ to the transparent conducting oxide (TCO). The band gap of GO72 is apparently narrowed to allow charge carrier flows from the band gap of TiO_2_, resulting in efficient electron mobility into the TCO film (photoanode). The fill factor, *FF* of GO72–TiO_2_-based photoanode (0.64) is lowered compared with bare TiO_2_ (0.65). The shape of *J–V* curve of TiO_2_-based photoanode DSSCs appears to be square-shaped compared with the GO72–TiO_2_ obtained curve. Another factor affecting the reduction of *FF* value is the series resistance, *R*_*s*_ of GO72–TiO_2,_ which is lower compared with the TiO_2_ sample. The mesoporous TiO_2_-based photoanode only achieves 2.96% because of its wide band gap energy and high charge recombination rate [25]. GO72 loading could reduce the charge transport resistance between the dye and TiO_2_ at the GO72–TiO_2_/N–719 dye/KI electrolyte interface, leading to the enhancement of overall PCE performance [26].

**Table 5:** Typical photovoltaic performance of DSSCs based on mesoporous TiO_2_ and TiO_2_ decorated with GO72.

Sample	Short Circuit Current Density, *Jsc* (mA/cm^2^)	Open Circuit Voltage, *Voc* (V)	Maximum Current Point, *Imp* (Amp)	Maximum Voltage Point, *Vmp* (V)	Fill Factor, *FF*	Photoconversion Efficiency (PCE), *ɳ* (%)
TiO_2_	8.02	0.57	6.42	0.46	0.65	2.96
GO72-TiO_2_	14.78	0.66	12.42	0.50	0.64	6.25

**Fig 8:**
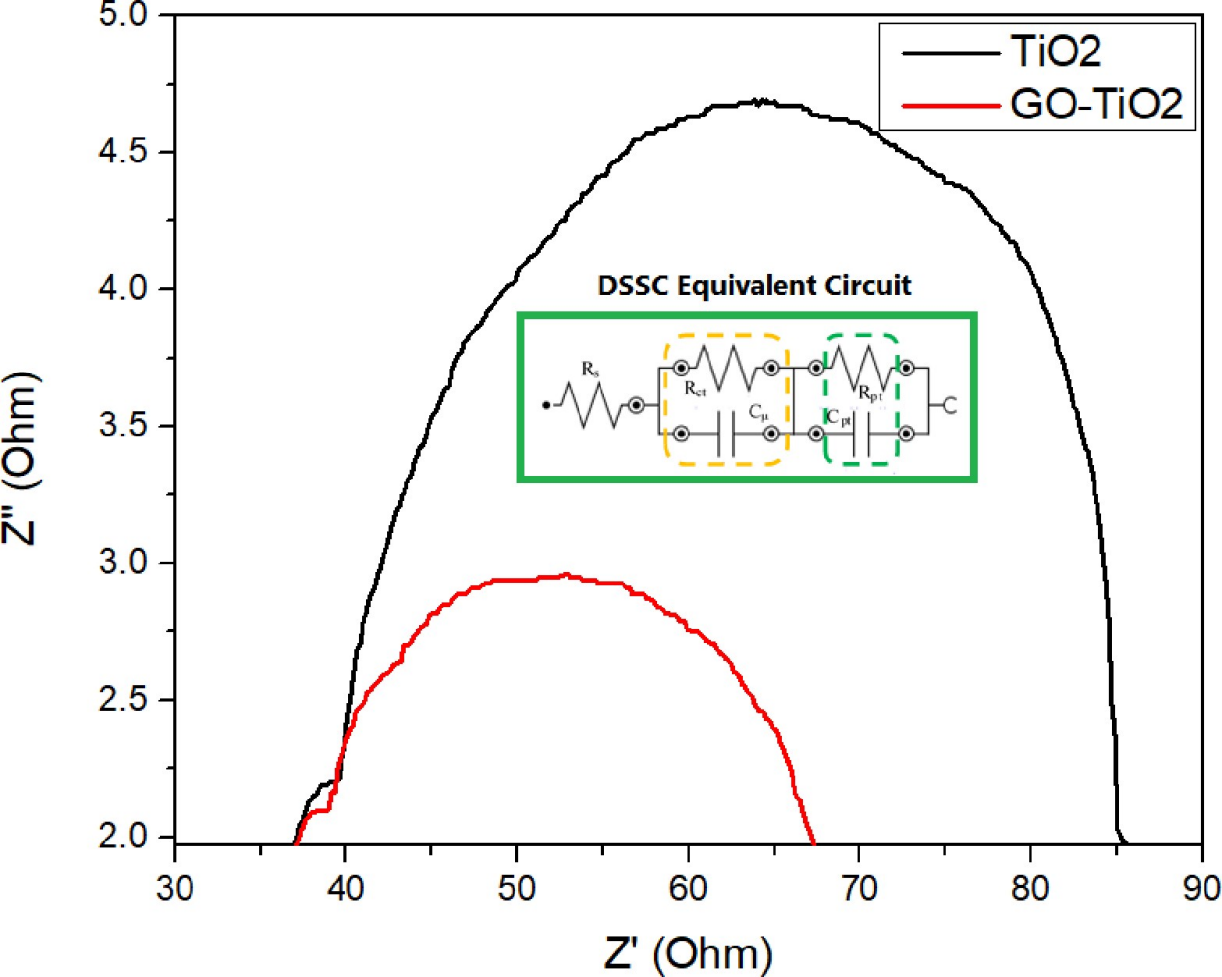
Nyquist plot and equivalent circuit of DSSCs (TiO_2_ and GO-decorated photoanode) from EIS measurements.

EIS analysis was applied to evaluate the interfacial properties, internal resistance, and charge-transfer kinetics of TiO_2_ and GO layers decorated with TiO_2_ in DSSCs [27]. EIS measurements were conducted under 100 mW/cm^2^ with a frequency range of 1–10^5^ Hz. The Nyquist plots of the DSSCs is tabulated in Fig 8. As shown in the Nyquist plot, each graph consists of two main semicircles to complete the electron flow cycle (small curve ~37–40 Ω; large curve ~40–86 Ω). For the first minor semicircle, the high-frequency region affected by charge transfer/photoexcited electrons is processed at the photoanode part. The next semicircle (middle-frequency region) is attributed to the TiO_2_/N-719 dye/KI electrolyte/Pt counter electrode or GO72–TiO_2_/N-719 dye/KI electrolyte/Pt counter electrode interface. The full range frequency of semicircles for DSSCs was fitted into the equivalent circuit shown in the inset of Fig 8. The equivalent circuit was divided into three parts, and the entire elements were series resistance (*R*_*s*_), which is represented as the first part (FTO/KI electrolyte), followed by the parallel circuit of charge recombination resistance (*R*_*ct*_) and chemical capacitance (*Cμ*), which are fitted for N-719 dye/GO72–TiO_2_/KI electrolyte, and final part were charge transfer resistance (*R*_*ct*_) with interfacial capacitance (*C*_*pt*_) as the KI electrolyte/Pt/FTO interface, which is connected in parallel with the low-frequency region. The value of *R*_*ct*_ obtained from the fitted equivalent circuit is used to measure the electron lifetime (*τ*) and tabulated in Table 6.

**Table 6:** Summaries of electron lifetime of TiO_2_-based and GO-decorated photoanode.

Photoanode Materials	R_ct_ (Ω)	C_μ_ (μF)	τ (ms)
TiO_2_	13.8	1105	15.2
GO72-TiO_2_	9.7	2967	28.8

As shown in Fig 8, the GO72–TiO_2_ photoanode-based DSSCs provides a lower curve than that of bare TiO_2_ photoanode-based DSSCs. This condition is because of the low charge recombination behavior with the addition of GO catalyst. In other words, GO could improve the charge electron loading rate and provide high transparency to receive efficient light. The TiO_2_-decorated GO film could enhance the photocatalytic activity and rapidly inject photoexcited electrons from the excited dye (S*) to the CB of GO72–TiO_2_ film.

As shown in Table 6 the R_ct_ of TiO_2_-decorated GO have smaller value compared with bare TiO_2_-based photoanode DSSCs because of the enhanced photocatalytic activity of GO film at dye/I_3_^−^ electrolyte, consequently leading to efficient charge carrier transport mobilities [28]. The presence of GO film improves the photoelectric performance through its intrinsic conductivity, consequently decreasing the *R*_*ct*_ value of photoanode film and resulting in higher lifetime of electron rate [29]. The lifetime, *τ* value, is obtained as follows:

$$\tau =C_{\mu }R_{ct}.$$ (3)

### Mechanism of DSSCs

**Fig 9:**
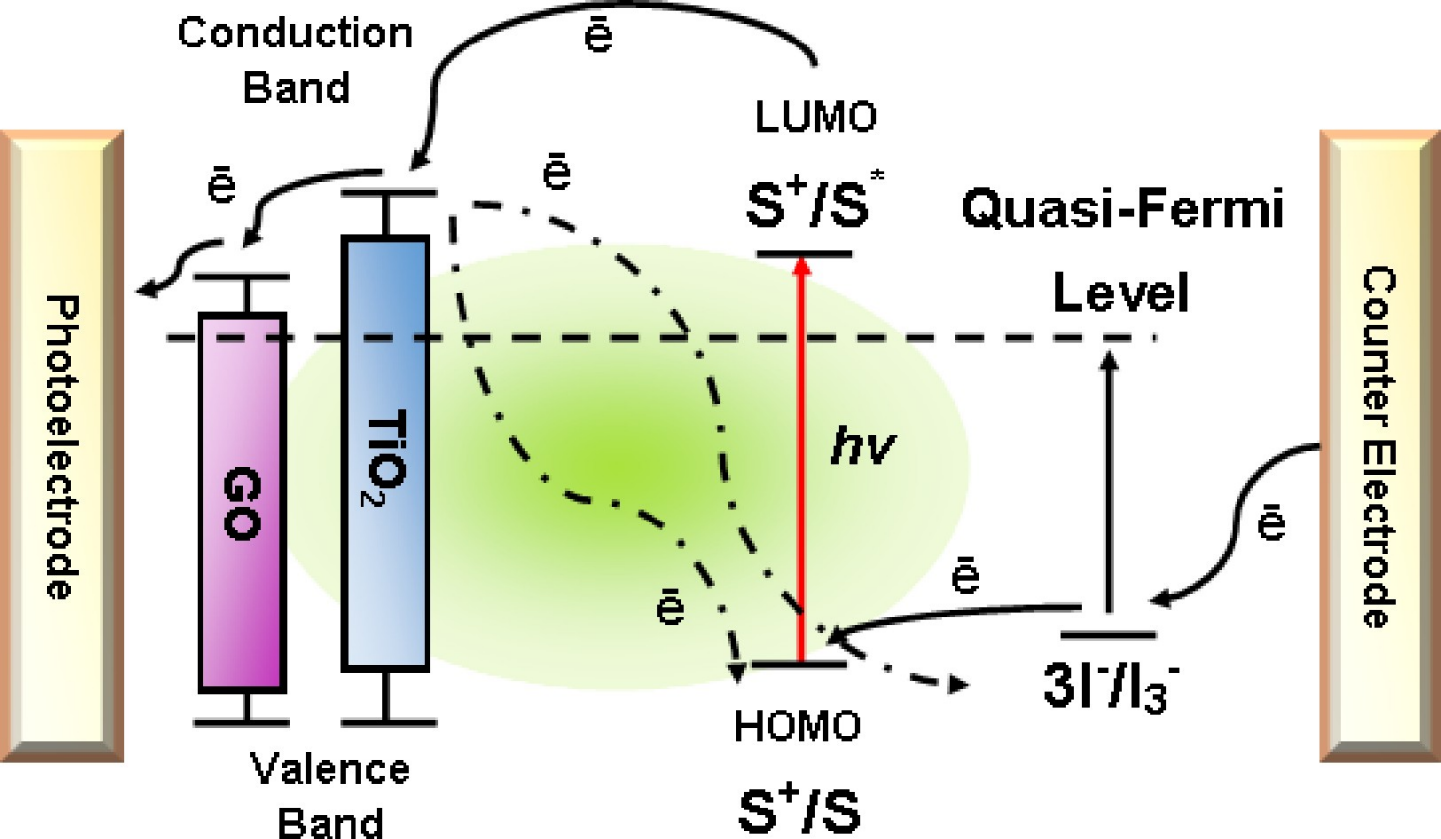
Working mechanism of DSSCs.

A working DSSC device is consist of photoanode coated on TCO/FTO glass slide deposited with a wide band gap semiconductor or metal oxide (TiO_2_, ZnO, Fe_3_O_4_, SnO_2_, etc.). DSSCs use a superconductor material, such as graphene, to improve the overall PCE performance. A dye-sensitizer is anchored on the metal oxide surface (N-719 dye, N-3, N-749, Z-907, etc.), an electrolyte is used for redox-coupled pair reaction (iodide and tri-iodide ion formation, *I*^*−*^*/I*_*3*_^*−*^), and the last element with FTO is coated with a metal thin film (typically platinum, silver, zinc, *etc*.) [30]. A complete schematic of a working mechanism of DSSCs is illustrated in Fig 9. During the irradiation of cell device under the UV light region, light photons are anchored with dye molecules (*S*) and consequently form the excited state S* after photon absorption. Within a short duration, the electron will be transported to TiO_2_, and S* is oxidized into *S*^*+*^ (Eq 4). At the same time, the electrons are injected into the CB of TiO_2_, transported through GO, subsequently flow to the external load, and reach the counter electrode. The oxidized dye molecules *S*^*+*^ is reduced into original *S* by regaining electrons from the organic electrolyte solution. Thus, the electrolyte solution contains *I*^*−*^*/I*_*3*_^*−*^ redox pair system, where the *I*^*−*^ ions are oxidized to *I*_*3*_^*−*^ ions and causing a hole in the dye molecules. To restore the *I*^*−*^ ions, a free electron is needed from the counter electrode to reduce the *I*_*3*_^*−*^ molecules into the *I*^*−*^ state (Eq 5). This complete loop of regenerating excitation/oxidation/reduction cycle is established for the continuous conversion of solar energy into useful electricity, which can be expressed as:

$$S+Photon\to S*(excited\;state)\to S^{+}+e^{-},$$ (4)

$$S^{+}+\frac{3}{2}I^{-}+e^{-}\to S+\frac{1}{2}I_{3}^{-}.$$ (5)

**Fig 10:**
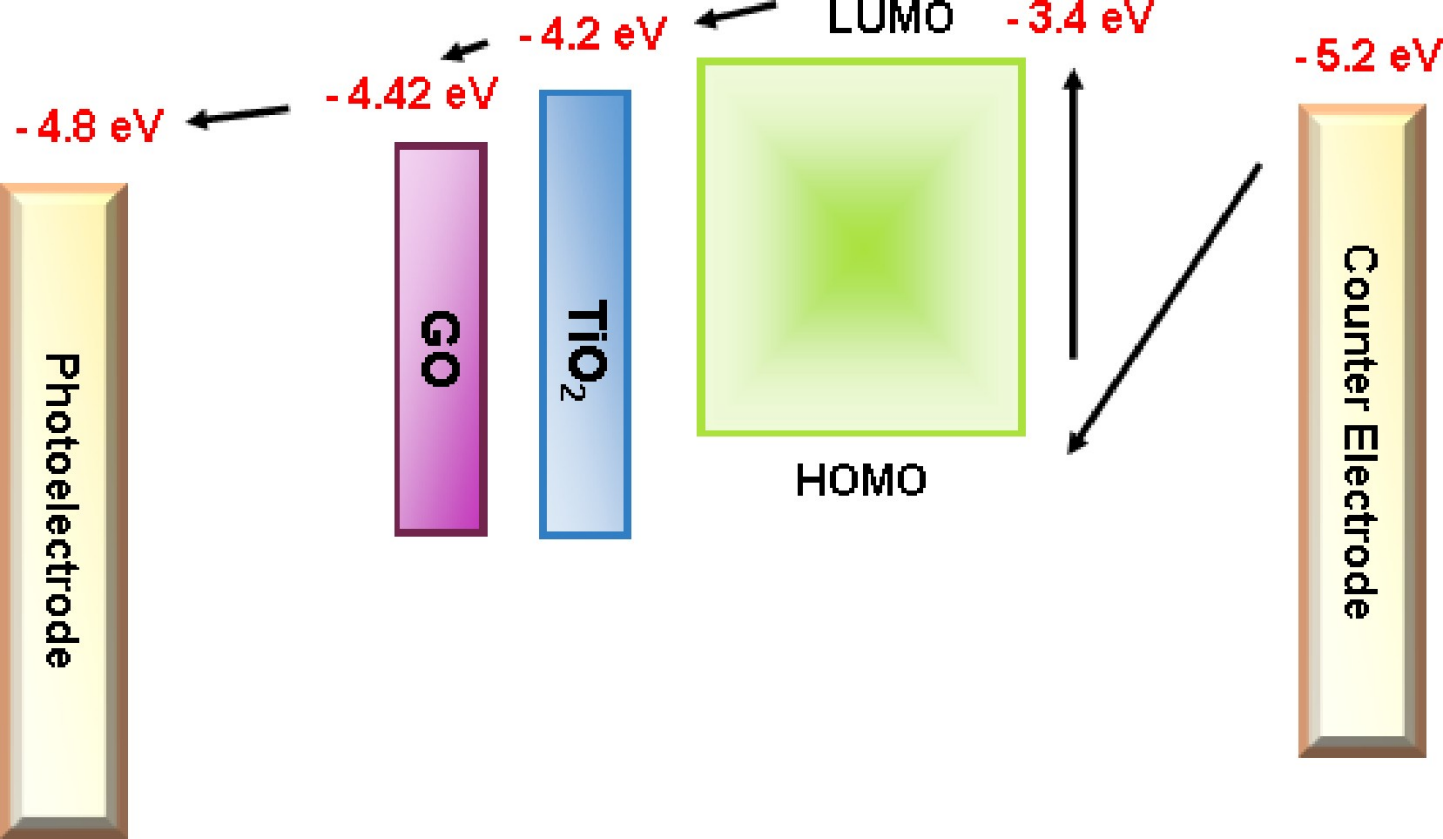
Electron energy level diagram of DSSCs.

Fig 10 shows the energy levels of the entire device configuration with photoanode/GO/TiO_2_/LUMO-HOMO energy level and counter electrode with −4.8, −4.42, −4.2, −3.4, and −5.2 eV. The excited electrons in dye molecules are introduced into a high energy state (HOMO) with associated energy level (~ −6.0 eV) and simultaneously create electron deficiency at the low energy state (LUMO, −3.4 eV). The LUMO energy level is higher than the CB of TiO_2_ in facilitating electron injection to GO thin film before transport to the FTO glass at the photoanode. GO was selected as the catalyst pathway with lower energy level (−4.42 eV) compared with TiO_2_ (−4.2 eV) because it relatively facilitates and accelerates electron charge carrier mobility, resulting in efficient PCE performance.

## Conclusion

In this study, GO was successfully synthesized using an improved Hummers’ method. The effects of stirring duration on the synthesis of GO were compared through several analyses, including XRD, FESEM, EDX, FTIR and Raman. GO with 72 h continuous stirring (GO72) at constant speed shows the optimum results. A large amount of oxygen functional groups appeared within the GO sample with controlled stirring duration of 72 h. GO72 was chosen, incorporated with TiO_2_, and tested for DSSCs. EIS analysis showed that GO72–TiO_2_ exhibits smaller charge- transfer resistance and higher electron lifetime compared with the TiO_2_-based photoanode. GO72–TiO_2_ demonstrates an optimized PCE result with 6.25% compared with pristine TiO_2_ with only 2.96%. This result suggests that GO72 creates a desired pathway for the DSSCs, thereby improving the absorption rate of dye at the photoanode.
